# The Histopathological Spectrum of Breast Lesions in Bangladeshi Women

**DOI:** 10.7759/cureus.103049

**Published:** 2026-02-05

**Authors:** Sabrina Razzaque, Md Ariful Islam, Tamanna Choudhury, Nadira Majid, Rahnuma Ahmad, Tanzeen Parmita Barsha, Zaida Bint Ershad, Mahmuda Abira, Mainul Haque

**Affiliations:** 1 Department of Pathology, Medical College for Women and Hospital, Dhaka, BGD; 2 Department of Pathology, International Medical College, Tongi, BGD; 3 Department of Pathology, Delta Medical College, Dhaka, BGD; 4 Department of Physiology, Medical College for Women and Hospital, Dhaka, BGD; 5 Department of Medicine, Medical College for Women and Hospital, Dhaka, BGD; 6 Department of Chemistry, The Radius International School, Dhaka, BGD; 7 Department of Physiology, Ibrahim Medical College, Dhaka, BGD; 8 Department of Pharmacology and Therapeutics, Eastern Medical College and Hospital, Cumilla, BGD; 9 Department of Research, Karnavati School of Dentistry, Karnavati University, Gandhinagar, IND

**Keywords:** accuracy, breast disease, breast fibroadenoma, breast lesions, clinicopathological correlation, early diagnosis, histopathology, inflammation, low- and middle-income countries bangladesh, malignancy

## Abstract

Background and objective

Breast lesions represent a wide clinicopathological spectrum, with breast cancer being the most common malignancy in Bangladeshi women. It often presents at a younger age and at an advanced stage, placing strain on the country’s limited oncology services. This study aimed to observe the histopathological pattern of breast lesions in women attending a tertiary care center in Dhaka and to identify clinical predictors of pathologically confirmed malignant breast cancer.

Methods

A retrospective, hospital-based study was conducted in the Department of Pathology, Medical College for Women and Hospital, Dhaka, from June to November 2025, including 160 women with histologically confirmed breast lesions. Clinical data and histopathology reports were analyzed using descriptive statistics, chi-square/Fisher’s exact tests, and multivariable logistic regression.

Results

The mean age was 31.79 ± 11.88 years, with most patients aged 30-39 years. The predominant diagnoses were fibroadenoma (52; 32.5%), granulomatous mastitis (24; 15.0%), invasive breast carcinoma (24; 15.0%), and fibrocystic change (13; 8.1%). Overall, 47.5% of lesions were benign, 35.6% inflammatory, and 16.9% malignant. Age ≥40 years (OR 2.582; 95% CI 1.754-3.802), bilateral involvement (OR 2.088; 95% CI 1.391-3.134), and a clinical impression of malignancy (OR 2.290; 95% CI 1.536-3.414) independently predicted malignancy. Histopathology-based classification demonstrated a sensitivity of 98.68%, a specificity of 74.07%, and an overall accuracy of 89.82% in differentiating benign from malignant lesions.

Conclusions

Benign and inflammatory lesions, particularly fibroadenoma and granulomatous mastitis, predominate among Bangladeshi women; however, a substantial malignant burden is evident, especially in older and bilaterally affected patients. Because the histopathology-based classification showed moderate specificity, further robust studies incorporating immunohistochemistry and molecular subtype analysis are warranted. Strengthening clinicopathological correlation and maintaining high-quality histopathology services are essential to support earlier diagnosis and targeted management in this setting.

## Introduction

Breast disorders comprise a diverse group of conditions, ranging from benign to malignant. They include inflammatory conditions, benign growths, atypical proliferations, and various carcinomas [1-3]. Inflammatory breast diseases include mastitis, abscesses, and granulomatous disease [4]. Noncancerous (or nonmalignant) tumors are commonly reported as fibroadenomas, fibrocystic changes, papillomas, phyllodes tumors, lipomas, and cysts [5]. In contrast, breast carcinoma includes invasive ductal or lobular carcinoma, ductal carcinoma in situ (DCIS), and rarer types such as medullary or mucinous carcinoma [6,7].

Diagnosis of breast disease involves imaging techniques, including ultrasound, mammography, MRI, and digital breast tomosynthesis [8,9], as well as pathology, including excision or needle biopsy followed by histopathology or cytology [10,11]. Common benign types, such as fibroadenoma, are often observed in younger women [12], while malignancy tends to peak later [13]. However, both benign and malignant lesions may present as palpable growths [14-16].

Breast diseases are widespread, with 60-80% of all breast tumors being benign [17]. This prevalence is particularly noted among women aged 30-50 years [12]. Fibroadenoma and fibrocystic changes are the most common benign breast growths, typically occurring in the age groups of 22-48 [18] and 20-50 years [12,19,20], respectively. One study reported that the mean ages of fibroadenoma and fibrocystic change were 23.1 and 31.1 years, respectively [21]. These benign breast tumors are generally detected as painful, palpable masses, often accompanied by redness and swelling, which is usually associated with mastitis. Fibroadenoma typically presents as a mobile, firm swelling [12], often located in the upper outer quadrant of the breast [5].

Noncancerous breast tumors are prevalent worldwide. In general, these benign growths are harmless and do not spread, unlike malignant tumors, and are therefore not life-threatening [12]. Nevertheless, certain types, such as papillomas or proliferative lesions, carry a slightly increased risk of developing breast malignancy, approximately 1.3-2.0 times that of healthy individuals [22,23], which necessitates regular monitoring [24]. In contrast, breast cysts or fibroadenomas often resolve spontaneously or require minimal medical or surgical intervention, thereby reducing morbidity [25]. Consequently, clinicians can focus more on symptom management, including lumps, pain, and emotional distress, because the risk of cancer cells spreading to distant organs is virtually negligible [26].

Globally, breast cancer is the most commonly diagnosed carcinoma among women [27], affecting every country and occurring at any age after puberty, with incidence rates increasing with age [28]. Rates are highest in high-income nations but are rising worldwide, including in low- and middle-income countries (LMICs) [29,30]. Breast cancer is frequently associated with lifestyle and hormonal factors, such as obesity, late age at first birth, and alcohol consumption, as well as marginalized socioeconomic status, which disproportionately affects underprivileged communities with limited access to healthcare, contributing to higher mortality in LMICs despite advances in treatment [31-34]. Key factors influencing breast cancer risk include obstetric events, breastfeeding practices, age at menarche, menopause, overall lifespan [35,36], environmental exposures [37], and the presence of highly penetrant genes, including BReast CAncer genes 1 and 2, phosphatase and tensin homolog, tumor protein p53, cadherin 1 (also known as E-cadherin), and serine/threonine kinase 11 (also called liver kinase B1) [38,39], with substantial geographical variation in incidence and mortality [3,27,28,40,41].

Breast cancer types are primarily classified as noninvasive or invasive. Noninvasive cancers are confined to the milk ducts, such as DCIS, an early-stage cancer that has not spread, or lobules, such as lobular carcinoma in situ, which involves abnormal cells in the lobules and is considered a risk factor rather than true cancer [42,43]. Invasive cancers occur when malignant cells spread beyond the ducts or lobules into surrounding breast tissue [44]. The most common invasive types are invasive ductal carcinoma, which originates in the ducts and spreads to surrounding tissue, and invasive lobular carcinoma, which arises in the lobules and expands into breast tissue [45]. Subtypes are further defined by hormone receptor status, human epidermal growth factor receptor 2 (HER2) expression, or triple-negative breast cancer, an aggressive form of invasive breast cancer [46]. Rare forms, accounting for 1-5% of cases, include inflammatory breast cancer [47-49].

Globally, breast cancer represents a major public health challenge, with an estimated 2.3 million new cases and 670,000 deaths in 2022 [50]. It contributes substantially to disability-adjusted life years [51], encompassing both years of life lost due to premature mortality and years lived with disability [51-54]. The disease negatively impacts quality of life [55], increases healthcare demands through both physical and psychological burdens [56], and imposes significant costs on public healthcare systems as well as out-of-pocket expenses [57-60].

Breast cancer mortality rates have declined in high-income countries due to improved screening and treatment [61-63]. However, it remains a leading cause of cancer-related death in developing countries, including low-income countries (LICs) and least developed countries, where survival rates are considerably lower [61,64-66]. In 2022, breast cancer was diagnosed in 2.3 million women worldwide, resulting in 670,000 deaths [28,67]. While overall mortality has decreased in some countries, for example, a 43% drop in the United States since 1989 [68], health disparities persist, with poorer outcomes in emerging economies and among disadvantaged or marginalized populations [31]. Early detection, improved healthcare access, and lifestyle modifications, addressing risk factors such as obesity and alcohol consumption, are associated with higher survival rates. In affluent regions, five-year survival exceeds 82%, whereas in LICs, it ranges from 12% to 46% [69-71].

Objectives of the study

Although multiple previous studies in Bangladesh have provided information on breast tissue characteristics and molecular subtypes of breast cancer cases, we aimed to conduct a more rigorous statistical analysis. This included logistic regression and an assessment of the sensitivity, specificity, and predictive value of histopathological examination to determine the current profile of breast pathological conditions among Bangladeshi patients.

Problem statement

A breast lesion is an abnormal mass or growth in breast tissue. Most breast lumps are noncancerous, such as cysts or fibroadenomas. However, some lesions may be premalignant or increase the risk of developing a subsequent malignant neoplasm [72,73]. These conditions are often described as “intermediate or gray lesions,” including C3 (atypical, probably benign) and C4 (suspicious, favor malignant) categories [74]. High-risk breast disorders encompass a diverse range of pathologies, some of which are considered preneoplastic lesions [75,76].

Breast pathological conditions are a significant public health concern in Bangladesh, where breast cancer is the most common malignancy among women, with a high prevalence in younger age groups (15-44 years) [77]. The average age of Bangladeshi breast cancer patients is 46.24 ± 7.4 years, with a peak incidence between ages 41 and 50. Additionally, 82.35% and 77% of patients were reported to have advanced breast carcinoma (Stage III or IV) and were under 50 years old [78]. Limited awareness of breast cancer in Bangladesh often results in delayed diagnosis at terminal stages [79,80].

To address this, public health institutions in Bangladesh, including the Ministry of Health and Family Welfare, the World Health Organization, and public postgraduate medical universities, have implemented strategies to increase participation in screening programs, such as clinical breast exams (CBEs), mammography, and patient monitoring and management programs. These initiatives focus on timely diagnosis, ongoing medical education for health professionals, and the development of national capacity for breast pathology research and therapeutic interventions [81-84].

## Materials and methods

Study design and ethical approval

This retrospective, hospital-based study was conducted at the Department of Pathology, Medical College for Women and Hospital, Dhaka, Bangladesh, using pathology laboratory records from June 2025 to November 2025. Ethical approval was obtained from the Institutional Review Board of the Medical College for Women and Hospital (approval MCWH/Ethical Committee/2025/18 (6), dated June 2, 2025).

Sample size and sampling

The required sample size was calculated using the single proportion formula:

$n = \frac{Z^2 \, p \, (1-p)}{d^2},$

where p is the estimated prevalence, Z is the Z-score for a 95% confidence level, and d is the absolute precision. A prevalence of p = 0.70 for benign breast disease among breast lesions was adopted from a large Asian epidemiological and histopathological study [85]. With a 95% confidence level (Z = 1.96) and an absolute precision of 7.5%, the minimum sample size was 144. Allowing for a 10% margin for exclusions and incomplete data, the final target sample size was set at 160, which was achieved in this study.

All eligible cases were enrolled consecutively from the pathology archives. Female patients of any age who underwent breast biopsy, lumpectomy, mastectomy, or other breast tissue excision and had a confirmed histopathological diagnosis were included. Cases were excluded if the tissue sample was inadequate for diagnosis, if the histopathology report was missing, or if essential clinical information (e.g., age or side of involvement) was incomplete. This consecutive, nonprobability sampling approach mirrors previous studies on the histopathological spectrum of breast lesions. A total of 160 women fulfilled these criteria and were included in the final analysis, providing a representative overview of benign, inflammatory, and malignant breast lesions encountered in routine tertiary care practice.

Data collection and statistical analysis

Data were entered into a spreadsheet and analyzed using IBM SPSS Statistics for Windows, Version 26.0 (Released 2018; IBM Corp., Armonk, NY, USA). Continuous variables, such as age, were summarized as mean ± SD, and categorical variables, including clinical diagnosis, histopathological diagnosis, and lesion categories (benign, inflammatory, and malignant), were presented as frequencies and percentages.

Associations between categorical variables (e.g., clinical type vs. pathological type, age group vs. malignancy status) were assessed using the chi-square test or Fisher’s exact test, as appropriate. Multivariable logistic regression was performed to identify independent factors associated with pathologically confirmed malignant breast cancer, with results expressed as ORs and 95% CIs. The diagnostic performance of histopathology-based classification (benign vs. malignant) was evaluated by calculating sensitivity, specificity, positive predictive value (PPV), negative predictive value (NPV), and overall accuracy. A two-sided p-value < 0.05 was considered statistically significant.

## Results

**Figure 1:**
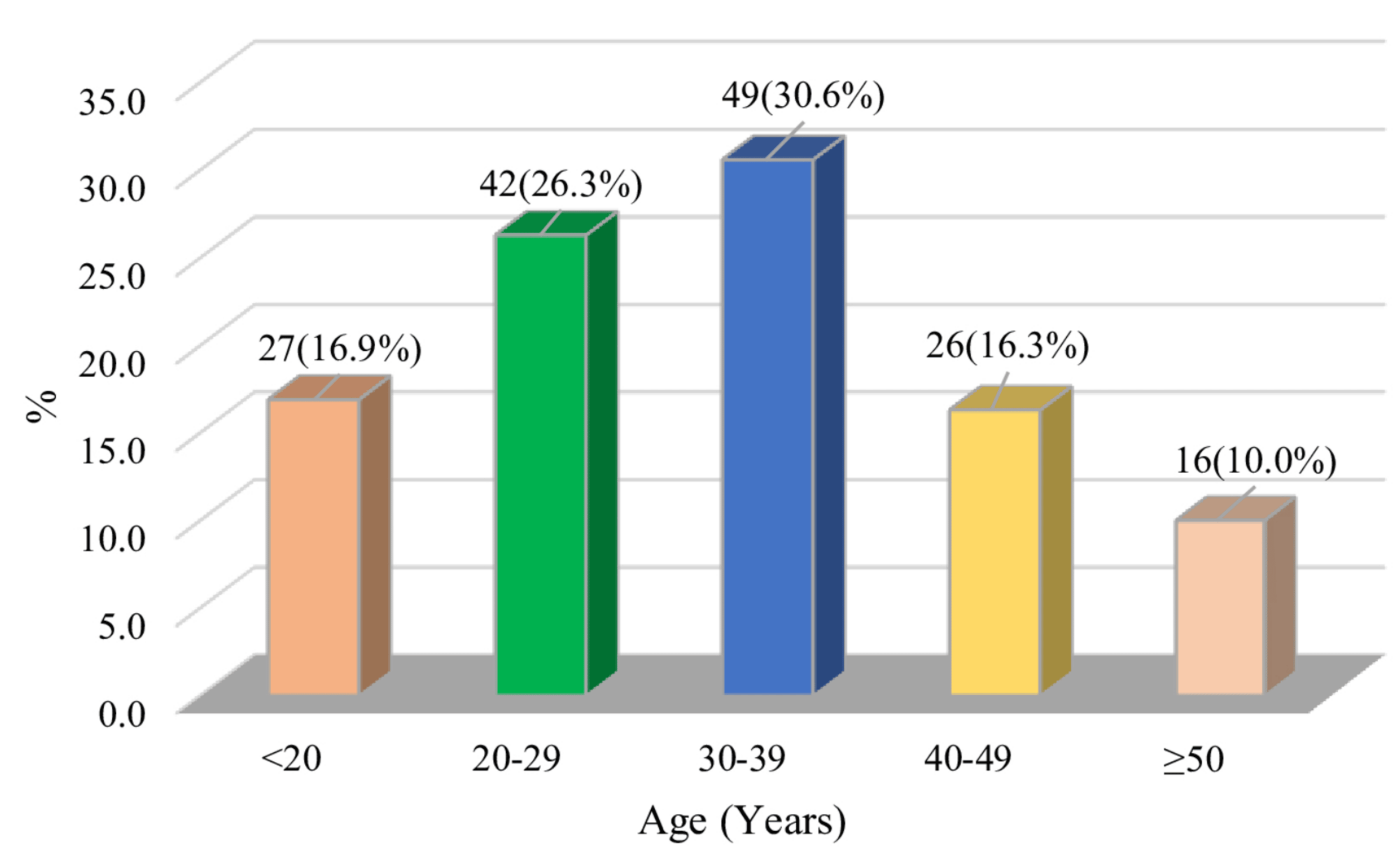
Distribution of study subjects by age (N = 160) Data are expressed as frequency, percentage, and mean ± SD. Illustration credit: Mahmuda Abira

In this study, the mean age of patients with breast lesions was 31.79 ± 11.88 years, with the majority, 49 (30.6%) subjects, in the 30-39 years age group (Figure 1).

**Figure 2:**
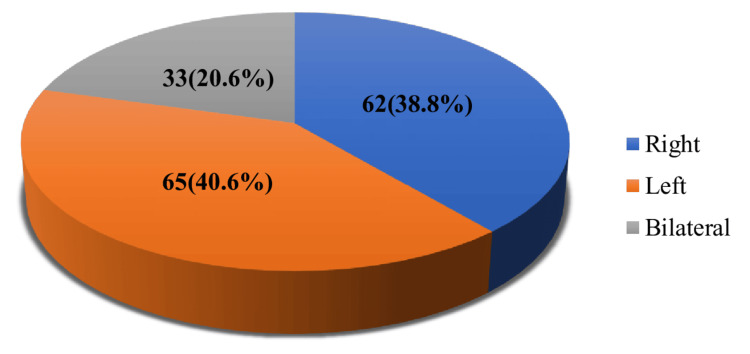
Distribution of study subjects by breast side involvement (N = 160) Data are expressed as frequency, percentage, and mean ± SD. Illustration credit: Mahmuda Abira

Regarding breast involvement, 65 (40.6%) females had left breast pathology, 62 (38.8%) had right breast pathology, and 33 (20.6%) had bilateral involvement (Figure 2).

Most respondents were clinically diagnosed with breast abscess (44, 27.5%), fibroadenoma (44, 27.5%), breast lump (23, 14.4%), and breast carcinoma (17, 10.6%) (Table 1).

**Table 1:** Distribution of study subjects according to clinical diagnosis (N = 160) Data are expressed as frequency and percentage.

Clinical diagnosis	Frequency	Percentage (%)
Breast abscess	44	27.5
Breast lump	23	14.4
Mastitis	11	6.9
Granulomatous mastitis	1	0.6
Fibroadenoma	44	27.5
Fibrocystic changes	5	3.1
Ulceration	1	0.6
Breast carcinoma	17	10.6
Ductal carcinoma	2	1.3
Infected galactocele	3	1.9
Intraductal papilloma	1	0.6
Keloid (skin lesion)	1	0.6
Paget’s disease	1	0.6
Phyllodes tumor	3	1.9
Suspected proliferative breast lesion without atypia	1	0.6
Suspected lump	1	0.6
Suspicious malignancy	1	0.6

**Figure 3:**
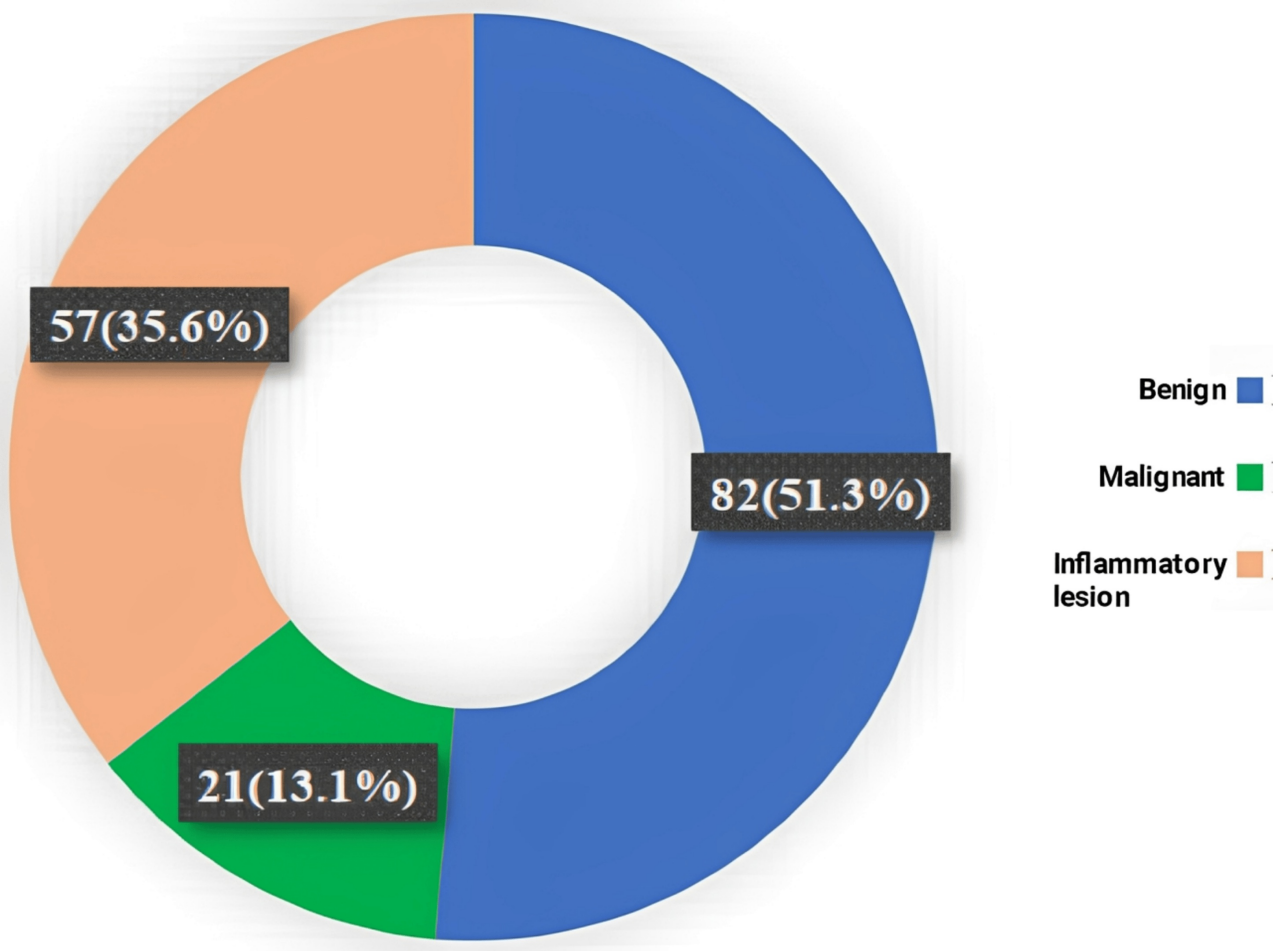
Distribution of study subjects according to clinical types (N = 160) Data are expressed as frequency and percentage. Illustration credit: Mahmuda Abira

Among the patients, 82 (51.3%) had benign lesions, 21 (13.1%) had malignant lesions, and 57 (35.6%) had inflammatory lesions (Figure 3).

Pathological diagnosis revealed fibroadenoma in 52 (32.5%) cases, granulomatous mastitis in 24 (15%), invasive breast carcinoma in 24 (15%), fibrocystic change in 13 (8.1%), and other lesions (Table 2).

**Table 2:** Distribution of study subjects according to pathological findings (N = 160) Data are expressed as frequency and percentage.

Histopathological diagnosis	Frequency	Percentage (%)
Abscess wall	2	1.3
Acute mastitis	9	5.6
Atypical ductal hyperplasia	1	0.6
Benign phyllodes tumor	1	0.6
Breast abscess	5	3.1
Chronic mastitis	5	3.1
Dermatofibroma	1	0.6
Fat necrosis	1	0.6
Fibroadenoma	52	32.5
Fibrocystic change	13	8.1
Galactocele	4	2.4
Granulomatous mastitis	24	15
Inflammatory breast carcinoma	24	15
Keloid (skin lesion)	1	0.6
Lipoma	1	0.6
Malignant phyllodes tumor	1	0.6
Mammary duct ectasia	1	0.6
Mammary Paget disease	2	1.3
No residual tumor (post-chemotherapy)	1	0.6
Periductal mastitis	1	0.6
Suppurative mastitis	10	6.2

**Figure 4:**
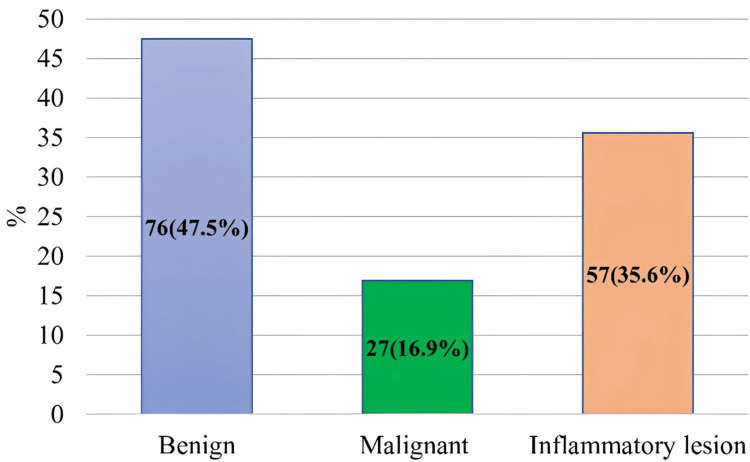
Distribution of study subjects according to pathological types (N = 160) Data are expressed as frequency and percentage. Illustration credit: Mahmuda Abira

Regarding pathological types, 76 (47.5%) females had benign breast growth, 27 (16.9%) had malignant breast cancer, and 57 (35.6%) had inflammatory lesions (Figure 4).

Of the 82 women diagnosed clinically with a benign tumor, 75 were confirmed pathologically as benign and seven as malignant. Conversely, of the 21 cases clinically diagnosed as malignant, 20 were confirmed as malignant and 1 as benign (Table 3).

**Table 3:** Comparison of clinical and pathological types of breast lesions (N = 103) Fisher’s exact test was performed.

Clinical	Pathological		Total	p-Value
	Benign	Malignant		
Benign	75 (98.7%)	7 (25.9%)	82	
Malignant	1 (1.3%)	20 (74.1%)	21	0.410
Total	76 (100%)	27 (100%)	103	

**Figure 5:**
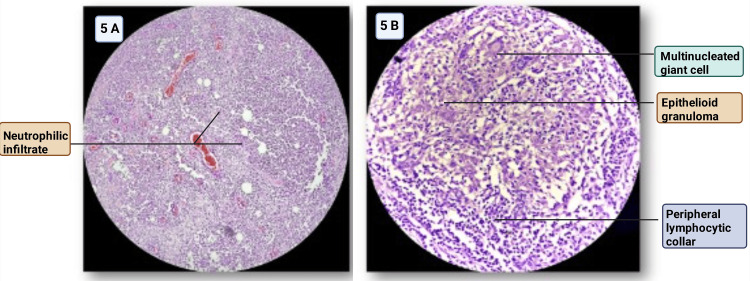
(A) Photomicrograph of suppurative mastitis showing sheets of acute inflammatory cells, predominantly neutrophils (H&E staining, ×100). (B) Photomicrograph of granulomatous mastitis showing epithelioid granulomas with peripheral lymphocytic collaring and multinucleated giant cells (H&E staining, ×200) Image credit: Sabrina Razzaque

Figure 5 shows the photomicrograph of breast mastitis.

**Figure 6:**
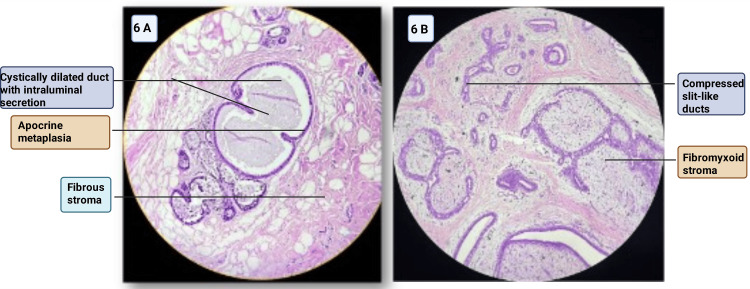
(A) Photomicrograph of fibrocystic change of the breast showing cystically dilated ducts lined by epithelium with apocrine metaplasia (H&E staining, ×100). (B) Photomicrograph of fibroadenoma showing compressed slit-like ducts within fibrous stroma (H&E staining, ×100) Image credit: Sabrina Razzaque

Figure 6A shows a photomicrograph of fibrocystic changes in the breast, and Figure 6B shows a photomicrograph of a fibroadenoma of the breast.

**Figure 7:**
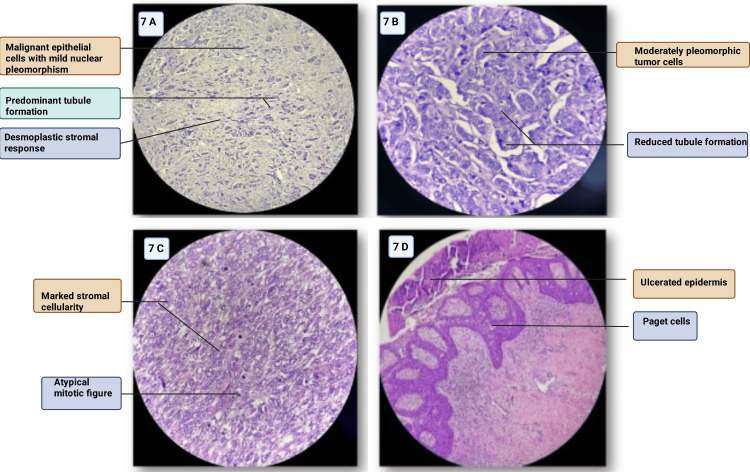
(A) Photomicrograph of invasive ductal carcinoma (NST), grade 1, showing predominant tubule formation with mild nuclear pleomorphism (H&E staining, ×100). (B) Photomicrograph of invasive ductal carcinoma (NST), grade 2, showing moderately pleomorphic tumor cells with reduced tubule formation (H&E staining, ×100). (C) Photomicrograph of malignant phyllodes tumor showing marked stromal cellularity and atypical mitotic figures (H&E staining, ×100). (D) Photomicrograph of Paget disease of the nipple showing large pale-staining Paget cells scattered within the epidermis (H&E staining, ×100) NST, no special type Image credit: Sabrina Razzaque

Figure 7 illustrates photomicrographs of carcinoma of the breast.

**Figure 8:**
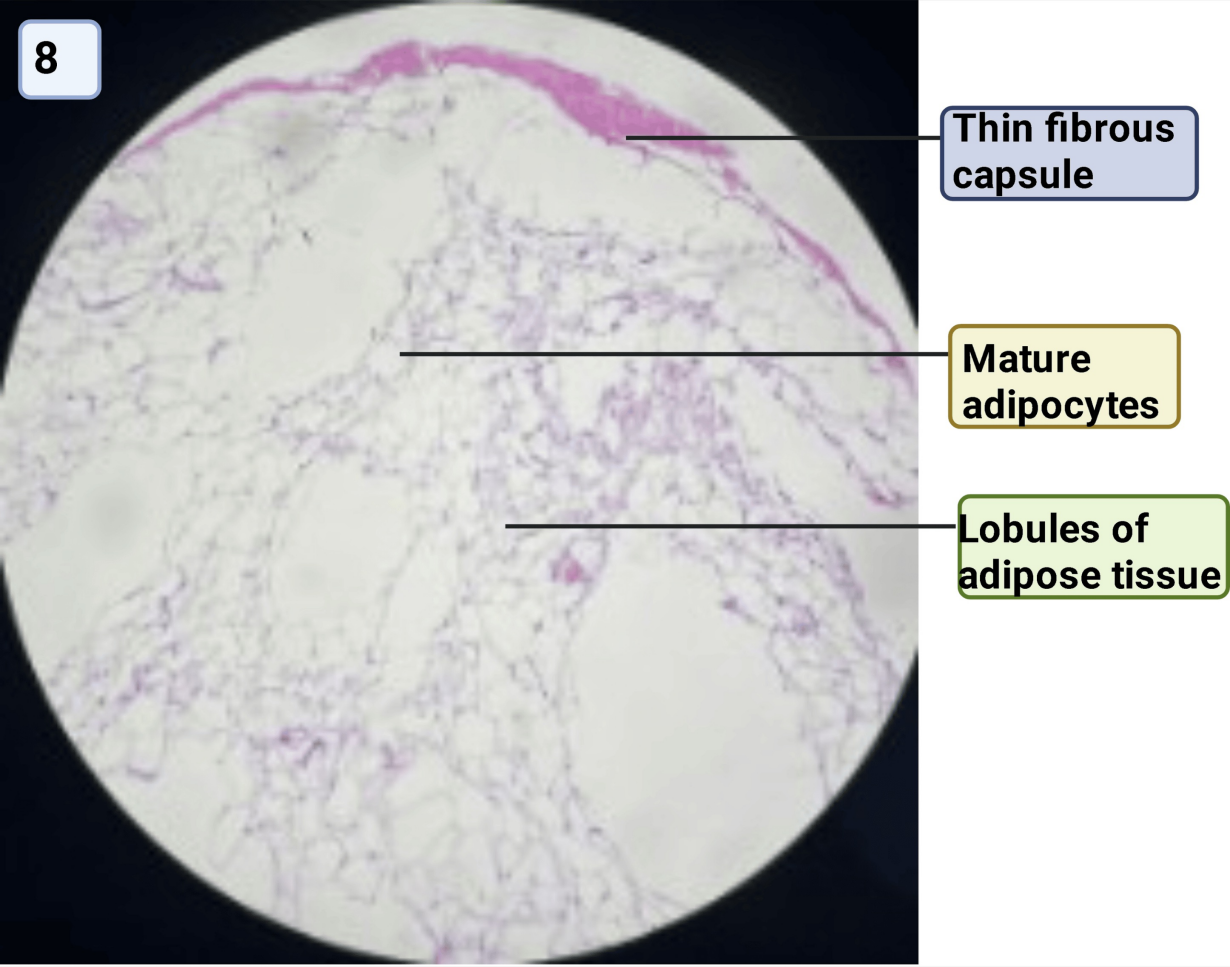
Photomicrograph of intramammary lipoma showing lobules of mature adipocytes (H&E staining, ×100) Image credit: Sabrina Razzaque

Figure 8 shows a photomicrograph of a breast lipoma.

In this study, multivariate logistic regression analysis revealed that age ≥40 years (OR: 2.582; 95% CI: 1.754-3.802; p < 0.001), bilateral involvement (OR: 2.088; 95% CI: 1.391-3.134; p < 0.001), and clinically diagnosed malignancy (OR: 2.290; 95% CI: 1.536-3.414; p < 0.001) were independently associated with pathologically confirmed malignant breast cancer (Table 4).

**Table 4:** Multivariate logistic regression analysis of risk factors for breast cancer (N = 103)

Variable	Frequency	OR	95% CI	p-Value
Age				
<40	61	2.582	1.754-3.802	<0.001
≥40	42	-	-	-
Side of involvement				
Unilateral	70	2.088	1.391-3.134	<0.001
Bilateral	33	-	-	-
Clinical diagnosis				
Benign	82	2.29	1.536-3.414	<0.001
Malignant	21	-	-	-

Our study showed that histopathology-based classification of benign and malignant breast lesions had strong sensitivity (98.68%) and moderate specificity (74.07%). It also demonstrated a PPV of 96.94% and an NPV of 89.82%, yielding an overall diagnostic accuracy of 89.82% for classifying breast cancer (Table 5).

**Table 5:** Diagnostic accuracy of histopathological findings for classifying breast cancer (N = 103) NPV, negative predictive value; PPV, positive predictive value

Statistic	Value	95% CI
Sensitivity	98.70%	92.9-99.9%
Specificity	74.07%	53.7-88.9%
PPV	87.10%	78.2-92.8%
NPV	96.90%	81.7-99.6%
Accuracy	89.80%	82.3-94.9%

## Discussion

Our study found that the mean age of Bangladeshi patients with breast lesions was 31.79 ± 11.88 years, with an age range of 30-39 years. Nahar et al. (2018) reported a mean age of 32.91 years and an age range of 11-70 years among Bangladeshi cases [86]. Their findings are broadly consistent with those of the present study, although the age range in their report was wider. This discrepancy may reflect increased awareness and better groundwork in Bangladesh over time. In our study, 40.6% and 38.8% of females had left- and right-sided breast pathology, respectively. Imran et al. (2025) found that breast pathology was primarily located in the left and right breasts at 49.8% and 49.2%, respectively, with a small proportion showing bilateral involvement [87,88]. Compared with these findings, our study observed no bilateral cases, and the prevalence on each side was slightly lower.

Most cases in our study were clinically diagnosed as one of four entities: breast abscess (27.5%), fibroadenoma (27.5%), benign growths (14.4%), and malignancy (10.6%). Kulkarni et al. (2016) reported that the most common nonmalignant breast pathologies included fibroadenoma, fibrocystic changes, and benign phyllodes tumors, with fibroadenoma accounting for 71.11% of cases [89]. These figures for noncancerous breast growths are considerably higher than those observed in the current study. Hammond et al. (2025) noted that breast malignancy is the most common carcinoma among women, constituting nearly one-third of all detected cancers [90]. The estrogen receptor (ER), a DNA-binding transcription factor, is expressed in 70-75% of all malignant breast neoplasms. Freihat et al. (2025) reported that, globally, breast malignancy was diagnosed in 2.3 million women, resulting in 670,000 deaths [28]. HER2 is a protein that promotes the growth of malignant cells; approximately 15-20% of breast cancers are HER2-positive, often resulting in more aggressive, rapidly growing tumors [91]. Progesterone receptor (PR)-positive breast carcinoma is characterized by tumor cells expressing receptors responsive to progesterone, occurring in 65-75% of cases. PR-positive tumors often indicate improved clinical outcomes and greater responsiveness to endocrine therapy compared with PR-negative tumors [92,93].

Among our study subjects, 51.3%, 13.1%, and 35.6% were clinically diagnosed with benign, malignant, and inflammatory breast disorders, respectively. These findings align partially with Aslam et al. (2013) [94], particularly regarding malignant breast growth. Similarly, Madubogwu (2020) reported that in Nigeria, less than 50% of breast lesions were neoplastic, with more than 50% being benign [95]. Histopathological diagnoses in our study included fibroadenoma (32.5%), granulomatous mastitis (15%), inflammatory breast carcinoma (15%), and fibrocystic change (8.1%). Razik et al. (2021) reported fibroadenomas in 53% of cases in Saudi Arabia, higher than in our study [96]. A Bangladeshi study by Amin et al. (2026) found that 81.63% of granulomatous mastitis cases were idiopathic and 18.36% were tubercular, which is higher than our reported prevalence [97]. Worldwide, invasive breast carcinoma is the most common malignancy among women, with a considerable lifetime risk of mortality, accounting for 11.7% (2.3 million) of newly diagnosed cases in 2020, and its incidence continues to rise [98]. Fibrocystic breast disorders are the most prevalent benign breast conditions, affecting up to 50% of women, particularly those aged 30-50 years, presenting with masses, pain, or cysts linked to hormonal fluctuations. Although generally benign, these patients require regular follow-up to rule out malignancy [12,99].

Our study confirmed that most breast disorders were benign, with a smaller subset being malignant, consistent with histopathological findings. These results align with an earlier study by Ibrahim et al. (2024) [100], which also reported that individuals older than 40 years are more likely to develop malignancies with bilateral involvement, a finding supported by multiple other studies [101,102].

Clinically diagnosed breast malignancies in our study were highly likely to be confirmed histopathologically, consistent with prior reports [103,104]. Histopathology-based diagnosis of breast carcinoma demonstrated strong sensitivity but moderate specificity, with a gap between PPV and NPV. These findings are comparable with previous publications [105,106]. Overall, the diagnostic accuracy of histopathology alone for classifying breast malignancies was limited, a result similar to Rakha et al. (2026) [107] but differing from DeVoe et al. (2024) [108].

Limitations

This retrospective, single-center study has several limitations. Conducted at a single center, the findings may not represent the diverse Bangladeshi population or rural settings. The sample size (n = 160) allows estimation of prevalence but limits subgroup analyses of rare lesions. Selection bias is present, as the study favors surgically excised cases, underrepresenting lesions managed through imaging or cytology alone. Retrospective data introduce documentation gaps, particularly regarding hormonal risk factors, and follow-up molecular profiling was not possible, limiting insights into subtypes. Additionally, our laboratory lacks advanced instrumentation necessary for confirmatory histopathological analyses.

Future research recommendations

Future studies should employ prospective, multicenter designs covering both urban and rural settings in Bangladesh to confirm these patterns in larger, more representative cohorts. Given the lower specificity observed in histopathology-based classification, more robust studies are needed to reinforce the role of histopathological examination for all breast lesions. Incorporating immunohistochemistry for ER, PR, and HER2 status, along with molecular profiling, would improve risk assessment and guide personalized treatment. Long-term monitoring of benign and inflammatory conditions, such as granulomatous mastitis, could provide accurate estimates of disease progression. Multidisciplinary approaches integrating clinical, imaging, and pathology data may streamline diagnostics. Additionally, increasing public awareness and expanding CBE programs could facilitate earlier detection. Establishing a national breast lesion registry would help track trends and inform health policy.

## Conclusions

This study highlights the spectrum of breast lesions observed among Bangladeshi women at a Dhaka hospital, with the majority being benign or inflammatory, alongside a subset of malignancies, particularly in younger women. The findings underscore the challenge of managing common breast conditions while identifying serious cases in resource-limited settings, where initial clinical diagnoses may be inaccurate but laboratory confirmation improves accuracy. The results are broadly consistent with global patterns but also reflect local challenges, including early-onset cases and delays in care. Our study identified low specificity in histopathology-based classification of breast lesions, indicating the need for larger-scale studies to improve diagnostic specificity. Enhancing laboratory capabilities, fostering physician collaboration, increasing public awareness, and implementing systematic screening can improve patient outcomes. Addressing these gaps promptly will strengthen breast health management in Bangladesh.
